# Enterococcus endocarditis in an infant with congenital renal duplex anomaly: A case report

**DOI:** 10.1007/s12024-026-01209-6

**Published:** 2026-04-06

**Authors:** Eteesha Rao, Srinivas Annavarapu

**Affiliations:** 1 Department of Medical Education, Medical School Newcastle University, Newcastle-upon-Tyne, NE1 4LP UK; 2 Department of Paediatric Histopathology, Alder Hey Children’s Hospital, Eaton Road, Liverpool, L12 2AP UK

**Keywords:** Enterococcus, neonatal sepsis, infective endocarditis, renal duplex anomaly, sudden unexpected infant death

## Abstract

Infective endocarditis is extremely rare in neonates and early infants but is associated with substantial morbidity and mortality. We report a sudden unexpected death in a 5-week-old male infant who was born at term in good condition. At one week of age, he presented with Enterococcus faecalis sepsis confirmed by blood culture. Ultrasound revealed a previously undiagnosed congenital renal duplex anomaly. Intravenous ampicillin-based beta-lactam antibiotics were administered after which the baby demonstrated clinical improvement. The baby was discharged after 2 weeks and was given antibiotic prophylaxis to be administered at home; blood culture was negative before discharge. At 5 weeks of age, he presented at the Accident and Emergency department in cardiac arrest. Despite aggressive resuscitation, the baby died. At autopsy, there was cardiomegaly and the aortic valves showed multiple friable vegetations (measuring approximately 2–3 mm), in keeping with infective endocarditis. Renal duplex anomaly was confirmed. Culture from heart blood, aortic vegetations and urine revealed a pure growth of Enterococcus faecalis. This case highlights the importance of early echocardiographic evaluation and renal anomaly surveillance in neonate with Enterococcus sepsis to prevent diagnostic delay and improve clinical outcomes.

## Key Points

Enterococcus neonatal sepsis can progress to infective endocarditis even after apparent recovery.

Congenital renal anomalies may act as persistent reservoirs promoting recurrent bacteraemia.

Echocardiography is essential following serious neonatal sepsis to exclude valvular infection.

Delayed diagnosis carries major clinical and forensic implications.

## Introduction

Infective endocarditis is the damage of the cardiac valves due to septic valvular vegetations resulting in significant morbidity and mortality. Although it is relatively rare in children and infants, the estimated incidence in neonates is 0.6–1.2 per 100,000 live births and it accounts for < 1% of paediatric infective endocarditis cases [1–3]. Most of the reported cases in infants and children are associated with underlying congenital heart disease or ***are*** related to catheter-associated vascular complications. Infective endocarditis in neonates and early infants is associated with a very high mortality rate, with many cases only identified at post-mortem due to the nonspecific nature of clinical manifestations [1–4].

 Enterococcus faecalis is a recognised but relatively uncommon pathogen in neonatal infective endocarditis compared with Staphylococcus aureus and Candida species [5–9]. This case demonstrates a rare but clinically significant association between Enterococcus faecalis sepsis, congenital renal duplex anomaly and fatal infective endocarditis. This infection appears community-acquired, as the infant was not hospitalised prior to the initial septic presentation.

## Case history

This case describes a sudden unexpected death in infancy of a 5-week-old male infant who was born at term in good condition. The pregnancy was complicated by gestational diabetes.

In the first four days of neonatal life, the baby fed normally and showed no significant health concerns. On day five of life, the baby stopped feeding and his skin appeared grey and mottled. The family members became concerned with his symptoms of vomiting, irritability and restlessness and took him to the hospital. Ultrasound scan revealed ***bilateral*** congenital renal duplex anomaly that was not picked up on the antenatal scans. Blood culture confirmed Enterococcus faecalis sepsis.

Ampicillin-based beta-lactam antibiotics (150 mg/kg/day) was administered intravenously and the infant demonstrated clinical improvement with stabilisation of vital parameters. The baby was discharged after two weeks and was given antibiotic prophylaxis to be administered at home. Repeat blood cultures prior to discharge confirmed clearance of bacteraemia. Clinical stabilisation was supported by improved feeding, haemodynamic stability and resolution of septic clinical features*.*

Echocardiography was not performed during this admission, representing a missed opportunity to exclude developing infective endocarditis in a high-risk neonate. The infant initially remained well at home following discharge. However, at five weeks of age he deteriorated suddenly, developing vomiting, diarrhoea and cessation of feeding. Despite urgent attendance at the Accident and Emergency department, he suffered cardiac arrest and died despite aggressive resuscitation. A post-mortem examination was therefore authorised.

### Timeline Summary

Day 0: Term birth in good condition.

Day 5: Enterococcus sepsis diagnosed; renal duplex anomaly detected.

Days 5–19: Intravenous ampicillin therapy administered.

Prior to discharge: Repeat blood cultures negative.

Week 5: Sudden clinical deterioration, cardiac arrest and death.

## Autopsy findings

**Fig. 1:**
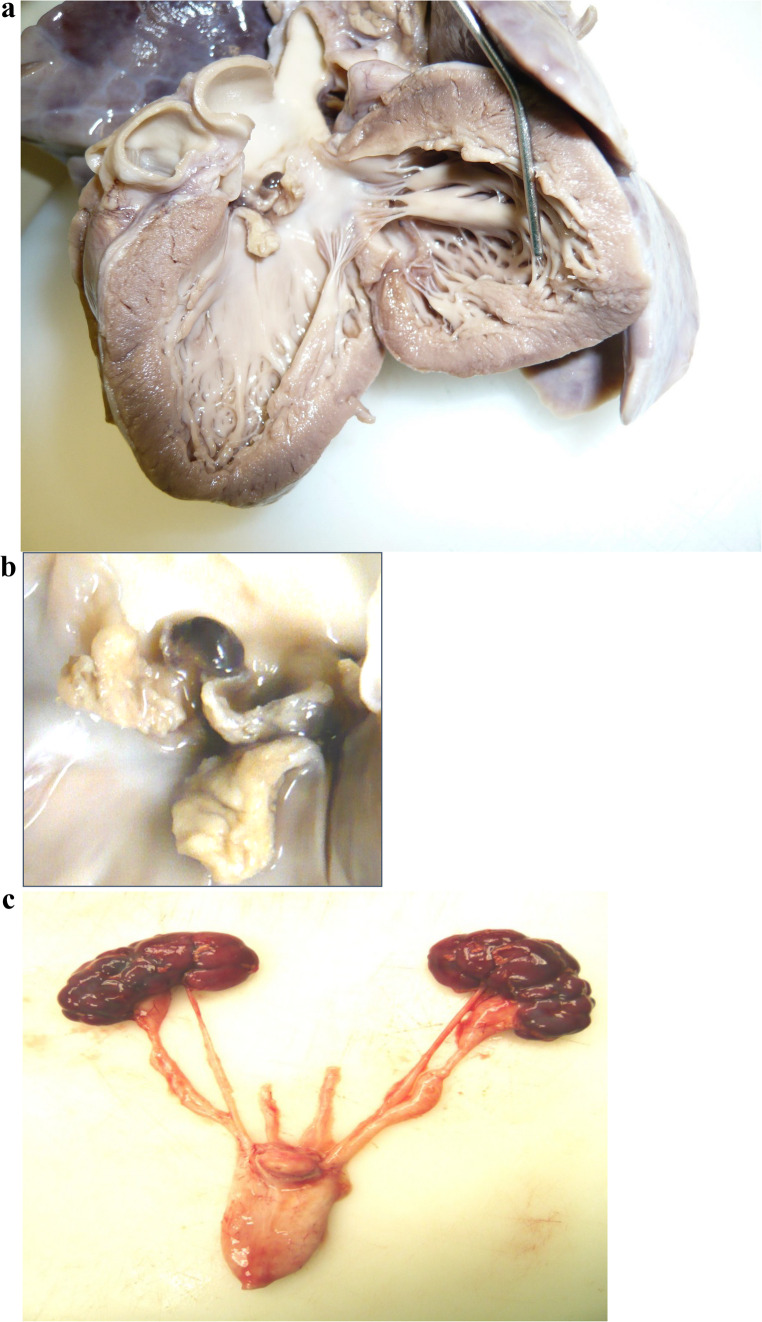
**a**. The aortic valve shows marked destruction, shortening and deformation of the cusps with incomplete closure. The vegetations are friable, irregular and grey-yellow (2-3mm 25mm across) covered with fibrin. Culture of the vegetations isolated Enterococcus faecalis. **b**. Closer view of the damaged aortic valves with infective endocarditis. **c**. The kidneys show prominent fetal lobulations. The renal pelves are dilated and show bilateral hydro-ureter. Both kidneys show renal duplex anomaly.

At autopsy, the body weight and linear body measurements were consistent with 5 weeks of age. The heart was enlarged (34 g; expected 23–26 g) but there was no septal or ventricular hypertrophy. The mitral valve was normal, however the aortic valve showed marked destruction, shortening and deformation of cusps with incomplete closure. Friable irregular grey-yellow vegetations (measuring approximately 2–3 m*m)* were present, covered with fibrin (Fig. 1a, b). There was associated aortic incompetence with left ventricular dilatation. Microscopic examination demonstrated inflammatory changes consistent with infective endocarditis; bacterial colonies were identified within the vegetations.

The kidneys (50 g; expected 34–38 g) were enlarged with pelvic dilatation, urinary stasis changes, bladder wall thickening and mild cortical atrophy (Fig. 1c). Mild cortical atrophy was present. The appearances were consistent with duplex kidneys. Culture from heart blood, aortic vegetations and urine revealed a pure growth of Enterococcus faecalis. Other organs were within normal limits. There was no macroscopic evidence of embolic infarction in the brain, spleen or kidneys.

## Discussion

Infective endocarditis is rare in neonates but carries significant clinical risk because of rapid progression to valve destruction, severe regurgitation and cardiovascular collapse [1–4]. Diagnosis is often difficult because symptoms commonly resemble severe neonatal sepsis, and classical peripheral signs seen in older individuals are typically absent.

Most cases in this age group are associated with congenital heart disease, catheter-related infection or neonatal intensive care exposure. Enterococcus faecalis is less common than Staphylococcus aureus or Candida in neonatal infective endocarditis but is clinically important due to its persistence in the bloodstream [3–5].

*I*n this infant, the congenital renal duplex anomaly provides a coherent explanation for the development of fatal infective endocarditis. Structural renal anomalies are known to predispose to urinary stasis. Urinary stasis encourages repeated urinary tract infection and persistent bacterial colonisation that in turn, increases the likelihood of recurrent bacteraemia. In early infancy, when immune defences are immature, recurrent bacteraemia increases the likelihood of bacteria adhering to cardiac valves. Once colonisation occurs, progressive bacterial proliferation produces friable vegetations, leading to structural destruction of the aortic valve, progressive haemodynamic deterioration and sudden cardiovascular collapse, as seen in this case.

 Gestational diabetes is recognised to increase the risk of congenital renal malformations, which further reinforces the importance of vigilant antenatal screening and structured clinical surveillance in such cases [10].

### Diagnostic delay and lessons learned

This case demonstrates the diagnostic challenge of recognising infective endocarditis early in neonatal sepsis. Failure to perform echocardiography following high-risk Enterococcus infection, absence of structured cardiac follow-up and reliance on negative blood cultures alone contributed to a missed opportunity for earlier diagnosis. These are clinically significant concerns in view of American Heart Association and European Society of Cardiology guidance recommending echocardiographic evaluation and prolonged monitoring in high-risk bacteraemia [11, 12].

### Medico-legal implications

This case raises important medico-legal learning considerations relating to adherence to standard-of-care investigation pathways in neonatal Enterococcus sepsis, the omission of echocardiography, the decision to discharge without cardiac imaging, adequacy of parental counselling regarding recurrence risk, antenatal detection of renal abnormality in diabetic pregnancy and documentation standards. These are presented as missed opportunities for earlier detection rather than allegations of negligence.

## Conclusion

Infective endocarditis, although rare in neonates, carries substantial mortality. Echocardiography should be routinely considered following Enterococcus sepsis, particularly in neonates with congenital anomalies or maternal diabetes. Early recognition, prolonged antimicrobial surveillance and vigilant renal monitoring may improve outcomes and prevent sudden unexpected infant deaths.

## Declarations

### Declarations

The authors have reviewed and edited the output and take full responsibility for the content of this publication.
